# A recombinant antibody platform for discovery and development of high affinity antibody pairs for diagnostic applications

**DOI:** 10.1002/alz70861_108859

**Published:** 2025-12-23

**Authors:** Ryan L Wallace, Sergei Bibikov, Troy Ganz, Haerin Park, Ava Bolandparvaz, Rebeca Meza, Katheryn Wetzel, Elsa P Ruble, Andreas Jeromin, April J Livengood, Kevin Harvey

**Affiliations:** ^1^ Aviva Systems Biology, San Diego, CA USA

## Abstract

**Background:**

The need for high affinity antibodies for the specific detection of biomarkers associated with neurodegenerative diseases is emerging as a priority for the development of high sensitivity assays to detect analytes in cerebral spinal fluid (CSF) and more importantly plasma or serum. High sensitivity platforms for ensuring detection at sub pg/ml levels have allowed for development of assays to a limited number of these biomarkers. Ultimately, though, the best and most important way to improve assay sensitivity is to identify high affinity binders up‐front. This enables robust assay development across a wide range of biomarkers and detection modalities including traditional plate‐based ELISAs.

**Method:**

We have developed a high‐throughput recombinant antibody development workflow leveraging AI‐influenced antigen design, single B cell cloning, and high throughput surface plasmon resonance (SPR) to identify high affinity binders. Additionally we utilize high‐throughput SPR screening for more rigorous specificity determination including post‐translational modifications. Furthermore, we are able to characterize putative binders through the assignment of epitope bins ensuring a diversity of epitopes for guided selection of the best sandwich pairs for immunoassay development.

**Result:**

Leveraging this workflow, we have identified high affinity picomolar binders for the detection of important neurodegenerative disease associated biomarkers in tissue, CSF and plasma including key biomarkers such as phosphorylated Tau (pTau) 217, pTau 181, pTau 231, and progranulin. We show that such an approach enables the identification of unique and novel antibody pairs that can improve assay sensitivity for detection in clinical disease samples versus controls in tissue, CSF, and blood.

**Conclusion:**

Though improvements in detection modalities have allowed the development of assays for the detection of low levels of disease‐associated blood‐based biomarkers, the identification of high affinity binders is the foundation for the development of robust assays displaying high sensitivity and specificity across all detection platforms or applications. Leveraging a high throughput recombinant antibody development pipeline to identify picomolar binders that display diverse epitope repertoires, we can accelerate development of clinically important assays for analytes that are not accessible with current diagnostic grade reagents or in more standard assay formats.

